# Unlimited Smartphone Data Plans in Older Adults With Data Deprivation: Quasi-Experimental Study

**DOI:** 10.2196/68930

**Published:** 2026-02-23

**Authors:** Seoyeong Choi, Eunjeong Choi, Suk-Yong Jang

**Affiliations:** 1 Department of Public Health, Graduate School, Yonsei University, 50 Yonsei-ro, Seodaemun-gu, Seoul, 03722, Republic of Korea, +82 2-2228-1511; 2 Department of Biohealth Industry, Policy Analysis Division, Graduate School of Transdisciplinary Health Science, Yonsei University, 50 Yonsei-ro, Seodaemun-gu,Seoul, 03722, Republic of Korea; 3 Department of Health Policy and Management, Graduate School of Public Health, Yonsei University, 50 Yonsei-ro, Seodaemun-guSeoul, 03722, Republic of Korea

**Keywords:** data accessibility, data deprivation, social participation, older adults, unlimited data plans, wellness

## Abstract

**Background:**

The growth of mobile health has underscored the critical importance of equitable internet access in promoting healthy aging. Among older adults, particularly those in digitally underserved populations, access to mobile data is often limited due to affordability and technological barriers, leading to a phenomenon known as “data deprivation.” This form of digital inequality limits the older adults’ ability to participate in social, recreational, and community-based activities, which are protective against isolation and decline in later life. In South Korea, where unlimited smartphone data plans have become increasingly accessible, a unique opportunity exists to examine the real-world association of improved data accessibility on older adults’ social lives and digital engagement.

**Objective:**

This study aimed to examine whether switching to unlimited smartphone data plans enhances social participation among older adults in South Korea. It also explored whether this relationship differs by demographic and socioeconomic characteristics, such as age, gender, region, household composition, and income level. The study focused on offline domains of social participation, including hobby gatherings, religious services, volunteering, and routine activities such as shopping.

**Methods:**

Data were drawn from the Korea Media Panel Survey (2016‐2022), a nationally representative longitudinal dataset. The final sample included 5021 individuals. Social participation was measured using a self-reported 8-item scale (8‐64 points) covering outdoor activities, volunteering, and more. A difference-in-differences approach was used to assess the association of switching from limited to unlimited smartphone data plans on social activity scores. Subgroup analyses examined heterogeneity by gender, household composition, income, and region.

**Results:**

Overall, among individuals aged 60 years and older, switching to an unlimited data plan was not associated with a statistically significant change in social activity scores. However, within this group, those aged 70 years and older showed a more notable—though not statistically significant—improvement (differential of 1.54, 95% CI −0.41 to 3.50). In contrast, older men living alone experienced a significant differential improvement of 6.44 (95% CI 3.39 to 9.50) points, compared with those who remained on limited plans.

**Conclusions:**

Although the overall association of unlimited data plans was limited, certain vulnerable subgroups—particularly older men living alone—experienced meaningful gains. These findings suggest that improving mobile data accessibility may enhance social engagement among digitally underserved older adults. Compared with more complex or resource-intensive interventions, expanding access to unlimited smartphone data plans may offer a relatively simple and scalable strategy to support healthy aging and reduce social isolation.

## Introduction

Across the Organization for Economic Cooperation and Development (OECD) countries, the population aged 65 years and older has significantly increased from under 9% in 1960 to 18% by 2021 [1]. The average of old age–related social expenditure (including pensions, health care, and long-term care) as a percentage of gross domestic product for OECD countries increased almost 1.5 times from 2000 to 2022, highlighting the growing economic burdens of an aging population [2]. In South Korea, the proportion of individuals aged 65 years and older is anticipated to reach approximately 40% by 2050, making it the fastest-aging nation among OECD countries [3]. Furthermore, South Korea has the highest older adult poverty rate among the 37 OECD member countries [4]. This significant aging issue necessitates effective policies to improve the lives of older adults. South Korea’s approach to this trend could provide valuable insights for other countries grappling with aging-related challenges.

The public health objective for older adults is to promote and maintain functional well-being, known as healthy aging. Successful aging requires preventing diseases, maintaining physical and mental function, and actively engaging in social participation, a principle endorsed by the World Health Organization (WHO) for national public health policies amidst population aging challenges [5]. Social scientists have long emphasized the importance of the relationship between social networks (active social participation) and health, arguing that social networks influence human health through 5 pathways: provision of social support, social influence, social participation and attachment, access to material goods and resources, and negative social interactions [6]. Social participation fostering healthy relationships is gaining attention as a concept of wellness and geriatric health [7]. The classification of social participation varies depending on the criteria, but generally aligns with the framework proposed by Berkman et al [8]. They noted that examples of social activity, known as social participation or engagement, may involve meeting friends, attending events, volunteering, fulfilling occupational duties, or participating in group recreational activities [8].

Previous studies have demonstrated a relationship between the quality of life, health outcomes of older adults, and social participation [9-11]. Specifically, reports indicate that older adult social participation is influenced by gender [12], region of residence [13], and household members [14]. However, traditional methods to enhance older adult social participation must evolve as society transitions into a digital era. Digital health is being highlighted as a mediator for this [15]. Studies have shown that higher smartphone use among older adults correlates with greater overall life satisfaction [16,17].

Older adults face a digital divide due to the lack of an environment that allows them to freely access the internet compared with other generations [18]. While prior research has largely focused on digital literacy, less attention has been paid to foundational access barriers—particularly mobile data affordability and availability [19,20]. Smartphone usage involves multiple dimensions, including device ownership, connectivity, and digital skills. However, access to stable and affordable data remains a prerequisite for all subsequent digital engagement. In this context, we introduce the concept of “data deprivation,” defined as the lack of consistent and affordable access to mobile data services. Unlike digital exclusion, which refers to general access to digital technologies, or “digital literacy,” which relates to skills and competencies, data deprivation emphasizes material constraints—such as limited data plans or high costs—that restrict older adults’ ability to participate online. We argue that this form of deprivation is a foundational driver of digital inequality, particularly among socioeconomically vulnerable older adults, and must be addressed before other digital inclusion efforts can be effective. Furthermore, data accessibility is not only essential for general social engagement but also for promoting digital health participation among older adults. In the context of mobile health (mHealth), which emphasizes the use of mobile devices to support public health and clinical care, access to mobile data accessibility serves as a bridge linking digital health technologies with real-world social engagement. Recent studies have shown that mHealth tools increasingly integrate features for mental wellness, social connection, and lifestyle tracking [21,22].

Without affordable and stable mobile internet accessibility, older adults may be unable to use health-related mobile apps, participate in online health communities, or benefit from remote monitoring technologies that support chronic disease management [23]. While previous discussions around older adult health have focused on economic or geographic access, this study brings attention to mobile data accessibility as a foundational but often overlooked determinant [24]. In this regard, our study aims to extend the discourse in digital equity by highlighting how mobile data accessibility, a prerequisite for mHealth use, may influence health-related behaviors such as social participation.

The relationship between internet use and social activity has long been debated through 2 opposing theoretical perspectives. The social reward hypothesis suggests that internet use enhances psychological well-being and social connectedness, especially among those with limited offline interactions [25]. In contrast, the displacement hypothesis argues that increased time online can replace face-to-face interactions, ultimately reducing social engagement and well-being [26]. While these theories have primarily been applied to younger populations, limited research has examined their relevance to older adults, who may have distinct digital usage patterns and social needs. While the social reward hypothesis has been the dominant perspective for adolescents prone to internet addiction [27,28], it may not be directly applicable to older adults. Most existing research has focused on reducing smartphone or internet use [29,30], with limited attention given to how these technologies might benefit older adults. As a result, it remains unclear which theoretical perspective—reward or displacement—better explains digital engagement among older adults, highlighting the need for empirical investigation in this population.

A unique aspect of the South Korean context is the widespread availability of unlimited smartphone data plans. Unlike expensive limited plans or Wi-Fi, which may pose cost or spatial restrictions, unlimited plans allow users to access the internet freely, regardless of time or place [31]. This feature offers a rare opportunity to examine whether removing mobile data constraints can enhance digital engagement and social participation among older adults. The observed effects in vulnerable subgroups suggest that improving mobile data accessibility could complement existing policies aimed at supporting social participation among older adults. Compared with complex and resource-intensive interventions, enhancing access to unlimited data plans could offer a relatively simple and scalable strategy. South Korea’s unique context—characterized by rapid population aging, high older adult poverty, and the widespread availability of unlimited data plans—provides an appropriate setting to examine the association between mobile data accessibility and social participation. Additionally, we examined how gender, residence, and household composition moderated the relationship between data accessibility and social participation in this context.

## Methods

### Data

In this study, we used data from the Korea Media Panel Survey (2016-2022). The Korea Media Panel Survey, constructed by the Korea Information Society Development Institute and funded by the Korean government, targets the same sample over an extended period to accumulate data [32]. This dataset aims to track changes in media environments and usage behaviors of households and individuals, providing fundamental data for in-depth research and policy development. We conducted face-to-face interviews with over 5000 households and individuals annually across 17 regions nationwide. The survey is divided into two main parts: one investigating media device ownership, subscription to broadcasting and communication services, and expenditure status; and the other examining life satisfaction, self-esteem, and similar factors.

### Study Population

In this study, data from 9788 participants in the 2016 Korea Media Panel Survey were used as baseline data. Participants who were already using a smartphone unlimited data plan in 2016 (n=1854) and those not present in both 2016 and 2022 (n=2396) were excluded. Participants with missing values in key variables such as smartphone plan type, social activity score, or baseline covariates (n=517) were also excluded. The final analytic sample consisted of 5021 participants.

### Variables

#### Social Participation

The dependent outcome in this study was social participation, for which the Korea Media Panel Survey used an 8-item self-report questionnaire [32]. Participants rated the frequency of their engagement in 8 types of activities on an 8-point Likert scale, ranging from “almost never” to “almost every day.” The activities included outdoor sports, social gatherings, volunteer and political activities, religious participation, hobbies, attending performances or exhibitions, watching sports, and outdoor shopping. These categories reflect the domains of social activity proposed by Berkman et al [8]. Total scores ranged from 8 to 64, with higher scores indicating more frequent social participation. This variable was only measured in 2016 and 2022.

#### Assessment of Unlimited Smartphone Data Plan

A smartphone unlimited data plan allows users to consume mobile data without any restrictions. Therefore, users can freely engage in activities such as web browsing, streaming, gaming, and social media. In this study, we use “data accessibility” to refer to the extent to which users can use mobile data without limitations such as data caps or additional charges. This is conceptually distinct from “data access,” which indicates the basic physical or technical availability of an internet connection or device. Based on changes in the unlimited smartphone data plan, which was the primary independent variable, the participants were divided into 2 groups: those who switched to an unlimited data plan in 2022 from a nonunlimited plan in 2016 (coded as “1”), and those who did not switch (coded as “0”).

### Other Covariates

To address the potential confounders that might influence both the independent and dependent variables, covariates were selected based on Berkman et al’s [8] social integration framework, which emphasizes how demographic and socioeconomic factors shape health through pathways such as social participation, access to resources, and social support. Additionally, variable selection was informed by prior empirical studies on social participation in later life, including Finlay and Kobayashi [33]. Specifically, we controlled for demographic characteristics (age, gender, educational attainment, marital status, employment status, household composition, and region) and socioeconomic status (household income quartile). Variables were categorized as follows: age groups (under 20, 20‐29, 30‐39, 40‐49, 50‐59, 60‐69, and ≥70 years); educational attainment (below middle school, high school, and college or higher); marital status (married, divorced or separated, and unmarried); employment status (unemployed, salaried employee, and self-employed or unpaid family worker); region (capital area, metropolitan, and city or province); household income quartiles; and household composition (living alone, living with a spouse only, and living with other household members excluding a spouse).

### Statistical Analysis

The data analysis was performed in 3 parts. First, to evaluate the covariate balance between the unlimited data plan and limited data plan groups, baseline characteristics were compared using standardized differences, with a difference of less than 0.1 (10%) typically considered insignificant. All variables were measured in 2016. For the subsequent analysis, only participants who did not use unlimited smartphone data in 2016 were selected and analyzed. Second, we applied a difference-in-differences (DID) regression model to estimate the effect of adopting unlimited smartphone data plans on social activity scores. The model included an interaction term between a treatment indicator (1=adopted unlimited plan by 2022; 0=remained on limited plan) and a time indicator (1=2022; 0=2016), while adjusting for baseline covariates such as age, gender, education, marital status, employment, income, household composition, region, and Wi-Fi access. SEs were clustered at the individual level. Third, a subgroup analysis was conducted for individuals aged 60 years and older, stratifying the sample by household composition, household income quartiles, and region, to explore whether the effects of adopting unlimited smartphone data plans differed across these demographic and household characteristics. Sampling weights were constructed following the procedures described in the Korea Media Panel Survey user guide, including initial design weights, nonresponse adjustment, and population benchmarking through raking ratio, as outlined in the panel survey’s methodological documentation. These weights were applied in all descriptive and regression analyses [34]. For baseline characteristics, we reported descriptive statistics and assessed between-group balance using standardized mean differences. CIs for regression estimates were obtained using robust standard errors clustered at the individual level. All statistical analyses were performed using SAS (Statistical Analysis System; version 9.4 M6; SAS Institute Inc).

### Ethical Considerations

This study used data from the Korea Media Panel Survey, a resource openly available for academic research. As the data do not contain identifiable private information, ethical approval was exempted by the institutional review board of Severance Hospital, South Korea (4-2024-0212). Informed consent was obtained from participants at the time of the original data collection, and the dataset was fully anonymized prior to analysis. No additional consent was required for this secondary data analysis. Participants’ privacy and confidentiality were protected through the use of deidentified data. No compensation was provided, as this study involved secondary analysis of an existing dataset.

## Results

**Figure 1.**
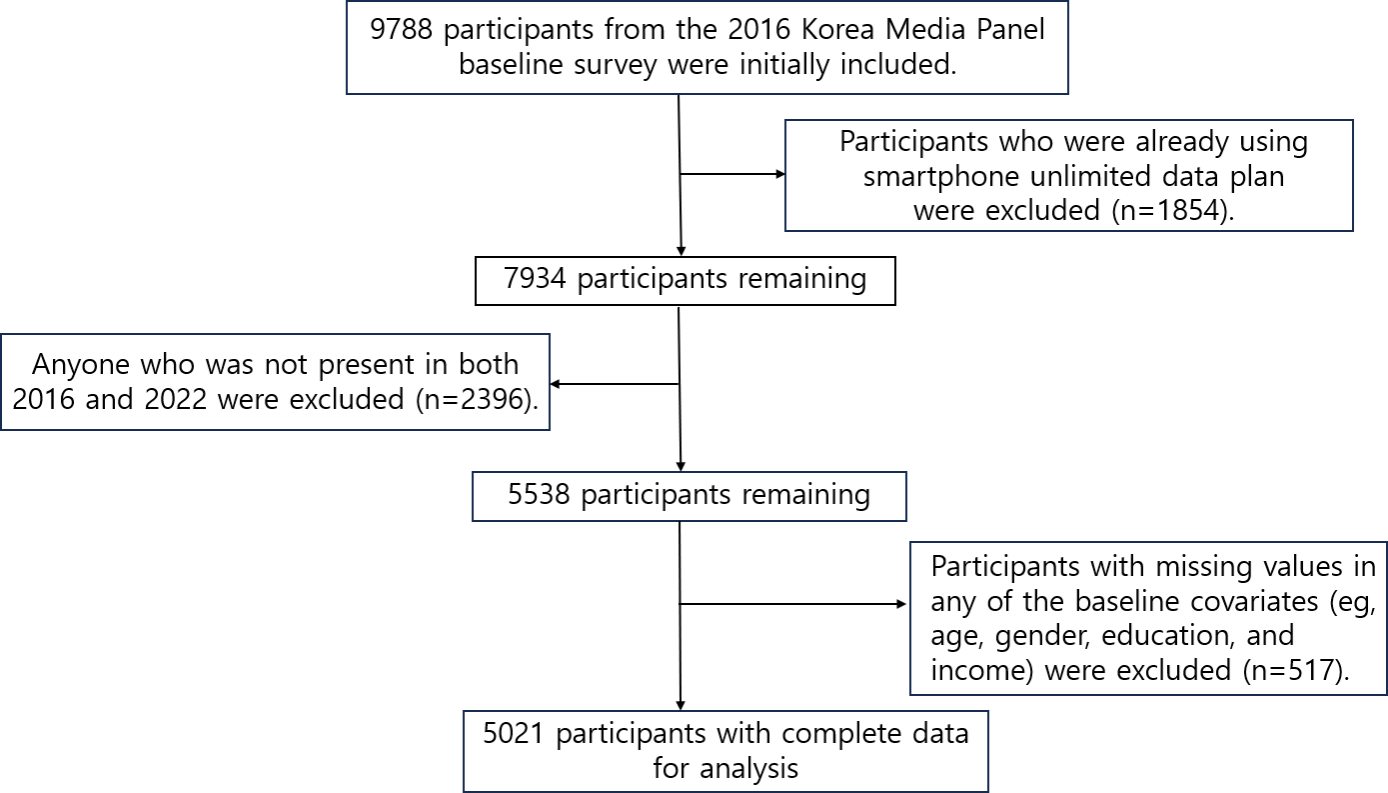
Flow diagram for study sample.

The final analytic sample consisted of 5021 participants with complete data (Figure 1). Table 1 presents the general characteristics of the study populations at baseline according to the smartphone unlimited data plan. Men outnumbered women among those who used unlimited data plans, who were mostly in their 40s and under 20s.

**Table 1.** General characteristics of study participants who did not use unlimited data plans at baseline (2016).^a^

Variables	Unlimited data plan^b^						
	Total (n=5021), n (%)		No→Yes (n=1404), n (%)		No→No (n=3617), n (%)		Standardized difference
Age (y)							0.907
<20	761 (15.16)		297 (21.15)		464 (12.83)		
20‐29	291 (5.80)		148 (10.54)		143 (3.95)		
30‐39	444 (8.84)		217 (15.46)		227 (6.28)		
40‐49	941 (18.74)		364 (25.93)		577 (15.95)		
50‐59	893 (17.79)		247 (17.59)		646 (17.86)		
60‐69	786 (15.65)		97 (6.91)		689 (19.05)		
≥70	905 (18.02)		34 (2.42)		871 (24.08)		
Gender							0.190
Man	2184 (43.50)		706 (50.28)		1478 (40.86)		
Woman	2837 (56.50)		698 (49.72)		2139 (59.14)		
Education							0.731
Under middle school	2073 (41.29)		294 (20.94)		1779 (49.18)		
High school	1707 (34)		562 (40.03)		1145 (31.66)		
College and higher	1241 (24.72)		548 (39.03)		693 (19.16)		
Marriage status							0.485
Married	3175 (63.23)		809 (57.62)		2366 (65.41)		
Divorced or separated	601 (11.97)		66 (4.70)		535 (14.79)		
Unmarried	1245 (24.80)		529 (37.68)		716 (19.80)		
Employment status							0.432
Unemployed	2578 (51.34)		645 (45.94)		1933 (53.44)		
Salaried employee	1619 (32.24)		614 (43.73)		1005 (27.79)		
Self-employed or unpaid family worker	824 (16.41)		145 (10.33)		679 (18.77)		
Household income							0.550
Low	2322 (46.25)		460 (32.76)		1862 (51.48)		
Mid-low	2000 (39.83)		664 (47.29)		1336 (36.94)		
Mid-high	587 (11.69)		233 (16.60)		354 (9.79)		
High	112 (2.23)		47 (3.35)		65 (1.80)		
Region^c^							0.238
Capital region	1612 (32.11)		540 (38.46)		1072 (29.64)		
Metropolitan	1288 (25.65)		377 (26.85)		911 (25.19)		
City or province	2121 (42.24)		487 (34.69)		1634 (45.18)		
Wi-Fi installation status^d^							0.455
Yes	2699 (53.75)		926 (65.95)		1773 (49.02)		
No	2322 (46.25)		478 (34.05)		1844 (50.98)		
Household composition							0.559
Single-person household	347 (6.91)		46 (3.28)		301 (8.32)		
Couple-only household	1226 (24.42)		166 (11.82)		1060 (29.31)		
Multigenerational household	3448 (68.67)		1192 (84.90)		2256 (62.37)		

a Values with unweighted frequency (weighted %) are presented. Standardized differences were used to compare baseline characteristics between groups. A value <0.1 was considered indicative of negligible imbalance.

b Unlimited smartphone data refers to a mobile plan offering unrestricted access to data usage without caps or limitations.

c The capital region of South Korea, encompassing Seoul and its surrounding metropolitan areas, serves as the political, economic, and cultural hub of the country, while metropolitan cities represent major urban areas and their environments, functioning as significant administrative and economic centers. City or province divisions in South Korea act as regional governmental bodies responsible for local governance and administration.

d Indicates whether Wi-Fi (wireless internet) is installed within the household.

Table 2 presents DID estimates of changes in social activity score before and after switching to an unlimited smartphone data plan by age group. Among people aged 60 years and older, the social activity score increased by 0.43 (95% CI −0.65 to 1.50; *P*=.44) points for those who switched from limited plans to unlimited plans between 2016 and 2022, though this change was not significant. When further stratified by age, individuals aged 70 years and older showed a numerically larger differential improvement of 1.54 (95% CI −0.41 to 3.50; *P*=.12) points. While suggestive, this finding was not statistically significant and should be interpreted with caution.

**Table 2.** Difference-in-differences estimates of changes in social activity score before and after using smartphone unlimited data plan.

Outcome	Unadjusted means (social activity score^a^)						Absolute difference in differences		
	Limited^b^^,c^→Limited			Limited^c^→Unlimited^d^			Adjusted^e^ (95% CI)	SE	*P* value
	Before^f^	After^f^	Difference	Before	After	Difference			
Age (y)									
<20	21.55	21.58	0.03	21.55	22.78	1.23	1.24 (–0.58 to 3.07)	0.93	.18
20-39	22.32	24.32	2.00	22.32	23.97	1.65	0.12 (–1.00 to 1.23)	0.57	.84
40-59	20.53	21.73	1.20	20.53	21.70	1.17	–0.44 (–1.19 to 0.32)	0.38	.25
≥60	17.16	17.83	0.67	17.16	20.06	2.90	0.43 (–0.65 to 1.50)	0.55	.44
60-69	17.99	19.65	1.66	17.99	20.16	2.17	–0.29 (–1.60 to 1.03)	0.67	.67
≥70	16.43	16.91	0.48	16.43	19.78	3.35	1.54 (–0.41 to 3.50)	1.00	.12

a Social activity score refers to the total combined score of 8 social participation and the maximum score is 64 points.

b Limited: smartphone limited data plan.

c The baseline excludes smartphone users with unlimited data plans.

d Unlimited: smartphone unlimited data plan.

e Adjusted for age, gender, education, marriage status, employment status, household income, region, Wi-Fi installation status, and family members.

f “Before” and “After” indicate the mean social activity score in the pre- and post-periods, respectively.

Gender-stratified subgroup analyses (Tables 3 and 4) indicated an increase in the estimated social activity score among the older adult group that switched to unlimited smartphone plans, stratified by independent variables (household composition, household income, and region). Particularly for older adult men living alone, the differential improvement was 6.44 (95% CI 3.39 to 9.50; *P*<.001) points higher for those who switched to unlimited data plans than those who remained on limited plans from 2016 to 2022. The estimate for socially isolated older men was higher in magnitude; however, as subgroup analyses are exploratory, these results should be interpreted with caution. Similarly, older adult men living in metropolitan areas were 3.47 (95% CI 0.15 to 6.78; *P*=.04) points higher than those who remained on limited plans, suggesting that differences in digital access across regions may affect the potential effectiveness of mobile interventions. For household income, differential improvement was observed in the low-income (DID change 1.54 points, 95% CI −0.05 to 3.60; *P*=.14) and high-income (DID change 7.67 points, 95% CI −3.03 to 18.38; *P*=.16) groups (suggesting that the change to unlimited plans had an association with social participation), but it was not significant. As shown in Table 4, the effects of several covariates were not as evident for older adult women as they were for older adult men. Among older adult women, the effects of unlimited data access were generally smaller and less consistent across subgroups. However, women living alone still showed a notable improvement of 3.38 (95% CI −0.55 to 7.30; *P*=.09) points, highlighting that living arrangement may be a more influential factor than gender alone.

**Table 3.** Gender-stratified subgroup analysis results by independent variables in male adults aged 60 years and older (n=1594)

Variables	Adults, n	Unadjusted means (social activity score^a^)						Absolute difference-in-differences		
		Limited^b^^,c^→Limited			Limited^c^→Unlimited^d^			Adjusted (95% CI)^e^	SE	*P* value
		Before^f^	After^f^	Difference	Before^f^	After^f^	Difference			
Household composition										
Single-person household (n=94)	94	17.70	17.11	−0.59	17.70	20.06	2.36	6.44 (3.39 to 9.50)	1.55	<.001
Multiperson household (n=1500)	1500	20.00	20.09	0.09	20.00	22.02	2.02	0.61 (−0.91 to 2.13)	0.77	.43
Household income										
Low	1034	18.16	17.46	−0.69	18.16	20.44	2.28	1.54 (−0.50 to 3.60)	1.04	.14
Middle	466	21.28	21.46	0.19	21.28	22.07	0.80	−0.05 (−2.74 to 2.63)	1.37	.97
High	94	22.62	21.24	−1.38	22.62	22.43	−0.19	7.67 (−3.03 to 18.38)	5.46	.16
Region^g^										
Capital region	371	20.09	20.94	0.84	20.09	23.27	3.17	3.47 (0.15 to 6.78)	1.69	.04
Metropolitan	367	20.76	21.08	0.32	20.76	21.55	0.79	0.03 (−2.64 to 2.71)	1.37	.98
City/province	856	19.28	18.67	-0.60	19.28	20.97	1.70	−0.31 (−2.20 to 1.56)	0.95	.75

a Social activity score refers to the total combined score of 8 social participation, and the maximum score is 64 points.

b Limited: smartphone-limited data plan.

c The baseline excludes smartphone users with unlimited data plans.

d Unlimited: smartphone unlimited data plan.

e Adjusted for age, gender, education, marriage status, employment status, household income, region, Wi-Fi installation status, family members.

f “Before” and “After” indicate the mean social activity score in the pre- and post-periods, respectively.

g The capital region of South Korea, encompassing Seoul and its surrounding metropolitan areas, serves as the political, economic, and cultural hub of the country, while metropolitan cities represent major urban areas and their environments, functioning as significant administrative and economic centers. City or province divisions in South Korea act as regional governmental bodies responsible for local governance and administration.

**Table 4.** Gender-stratified subgroup analysis results by independent variables in female adults aged 60 years and older (n=2321)

Variables	Adults, n	Unadjusted means (social activity score^a^)						Absolute difference in differences		
		Limited^b^^,c^→Limited			Limited^c^→Unlimited^d^			Adjusted (95% CI)^e^	SE	*P* value
		Before^f^	After^f^	Difference	Before^f^	After^f^	Difference			
Household composition										
Single-person household (n=567)	567	17.58	17.12	−0.47	17.58	21.25	3.67	3.38 (−0.55 to 7.30)	2.00	.09
Multiperson household (n=1754)	1754	19.95	20.27	0.31	19.95	22.24	2.29	−0.59 (−2.36 to 1.18)	0.91	.52
Household income										
Low	1671	18.16	17.71	−0.44	18.16	19.77	1.61	0.79 (−1.59 to 3.17)	1.21	.51
Middle	560	21.13	21.07	−0.06	21.13	21.95	0.83	−0.75 (−3.52 to 2.02)	1.41	.60
High	90	21.85	21.92	0.07	21.85	23.75	1.91	—^g^	—	—
Region^h^										
Capital region	542	19.99	21.14	1.15	19.99	23.61	3.62	−0.62 (−4.62 to 3.37)	2.04	.76
Metropolitan	517	20.75	20.33	−0.42	20.75	21.68	0.93	−0.03 (−2.82 to 2.77)	1.42	.99
City or province	1262	18.91	18.59	−0.32	18.91	20.87	1.97	0.70 (−1.47 to 2.86)	1.10	.53

a Social activity score refers to the total combined score of 8 social participation, and the maximum score is 64 points.

b Limited: smartphone-limited data plan

c The baseline excludes smartphone users with unlimited data plans.

d Unlimited: smartphone unlimited data plan.

e Adjusted for age, gender, education, marriage status, employment status, household income, region, Wi-Fi installation status, and family members.

f “Before” and “After” indicate the mean social activity score in the pre- and postperiods, respectively.

g Not applicable.

h The capital region of South Korea, encompassing Seoul and its surrounding metropolitan areas, serves as the political, economic, and cultural hub of the country, while metropolitan cities represent major urban areas and their environments, functioning as significant administrative and economic centers. City or province divisions in South Korea act as regional governmental bodies responsible for local governance and administration.

## Discussion

### Principal Findings

This study used a DID design to examine the potential association between unlimited smartphone data plans and changes in social participation of older adults. Notably, the findings were numerically larger among men and older adults living alone. These results suggest that unlimited data plans could be explored as a potential digital inclusion strategy for specific vulnerable subgroups.

Prior studies have primarily examined the relationship between improved digital literacy and increased social engagement among this population [16,17]. In contrast, our findings suggest that providing unrestricted smartphone access—regardless of digital literacy level—may be explored as a potentially meaningful intervention. This highlights the unique challenges faced by older adults who cannot freely use smartphones in the digital age. For instance, we found that living alone was significantly associated with lower social activity scores, indicating limited opportunities for social interaction compared with those living with others [35,36]. As a result, these individuals may depend more on digital tools for connection. The provision of unlimited data plans—by removing spatiotemporal restrictions on internet access—may reduce barriers to social participation for these individuals. While the small sample size limits the significance of the results, a notable differential change was observed in the incomes of those living in metropolitan areas. A previous study identified community-based activities as central to older adults’ social participation [30]. In rural or smaller communities, older adults often benefit from closer-knit social networks and community activities. In contrast, those living in metropolitan areas may face greater social isolation and thus rely more heavily on digital means for connection. This could explain why the provision of unlimited smartphone data showed a suggestive, numerically larger association with their social participation [37].

While no statistically significant overall effect was observed among individuals aged 60 years and older, subgroup analyses revealed a significant improvement in social activity scores for specific populations—most notably, older men living alone. These findings suggest that older adults’ ability to participate in social participation increasingly depends on access to digital devices and mobile data. For those with limited financial resources, the cost of data plans or smartphones may pose significant barriers, reflecting the broader economic inequality in digital participation. Gender-stratified analysis revealed that older men living alone experienced a numerically larger improvement in social activity following the adoption of unlimited smartphone data plans. This finding is particularly meaningful given that older men are generally less socially active than women [38], suggesting that enhanced digital access may help narrow gender disparities in social engagement. The effect was even more evident among male residents of metropolitan areas, indicating that digital connectivity plays a critical role in enabling social participation for this subgroup. The unlimited smartphone data plan differs from Wi-Fi. It reduces spatiotemporal constraints on internet use and encourages older adults to engage in social participation. Compared with traditional interventions such as community-based programs or digital literacy education [16,17], providing unlimited smartphone data plans offers a lower-cost and scalable alternative to enhance social participation among older adults. By reducing structural barriers to digital access, this approach could help address digital inclusion more directly and efficiently.

### Implications

These subgroup findings suggest that policymakers could consider subsidizing mobile data plans for vulnerable older populations, such as those living alone or in metropolitan areas. Conducted in South Korea—where smartphone penetration is high and unlimited data plans are relatively affordable—this study highlights how improved digital access can enhance social participation among older adults. The findings of this study may be relevant to other settings with high smartphone penetration, although their applicability will depend on local infrastructure, affordability, and broader socioeconomic context.

### Limitations

This study has some limitations. First, due to data source limitations, we analyzed data only from 2016 and 2022. Second, our analysis included only the covariates calibrated in this study, potentially omitting other unmeasured covariates that could influence the relationship between unlimited data use and social activity scores. Third, we lack precise information on the timing of participants’ transition to unlimited data plans. Therefore, we cannot pinpoint the exact time of participants’ switch to unlimited data and its subsequent association with their social activity scores over the 6-year period. To address this, we assumed an average switch point sometime in the middle years. In addition, we conducted multiple subgroup analyses to explore heterogeneous effects across demographic and household characteristics. While these analyses provide valuable insight, they also introduce the possibility of spurious findings due to multiple comparisons. Thus, subgroup results should be interpreted with caution and seen as exploratory.

In addition, we did not formally assess whether the assumptions of linear regression—such as normality of residuals, homoscedasticity, and absence of multicollinearity—were met. This may limit the robustness of our estimates and should be addressed in future studies. Finally, the data only included 2 time points (2016 and 2022), which limited our ability to verify the parallel trends assumption—an essential prerequisite for the validity of DID analyses. Although we aimed to minimize baseline imbalance and carefully selected covariates, the inability to test parallel trends remains a critical methodological limitation. Future research using panel data with multiple time points would allow for formal testing of this assumption and a more robust causal inference.

### Conclusions

This is the first study to use unlimited smartphone data plans to examine changes in social activity among older adults. By using longitudinal data with repeated measures of the same participants, we effectively captured changes in social activity over time. Therefore, our findings can inform policy discussions and contribute to the important public health goal of promoting social engagement among older adults.

This study unveils a significant association between older adults’ adoption of unlimited smartphone plans and their social engagement, suggesting that switching to an unlimited plan may be associated with increased social participation. Ensuring older adults have unrestricted internet access to electronic devices is crucial before initiating digital literacy initiatives. These findings highlight the potential of improving data accessibility as a supportive lever for promoting social participation in older adults, offering a comparatively simple and scalable approach alongside existing policy measures.

## Abbreviations

DID: difference-in-differences
mHealth: mobile health
OECD: Organization for Economic Cooperation and Development
SAS: Statistical Analysis System
WHO: World Health Organization

## References

[1] Raleigh VS (2019). Trends in life expectancy in EU and other OECD countries: why are improvements slowing?. https://www.oecd.org/content/dam/oecd/en/publications/reports/2019/02/trends-in-life-expectancy-in-eu-and-other-oecd-countries_93f16d1f/223159ab-en.pdf.

[2] Social expenditure database. Organization for Economic Co-Operation and Development.

[3] (2023). OECD health statistics. Organization for Economic Co‑operation and Development.

[4] (2023). Pensions at a glance 2023: OECD and G20 indicators. https://www.oecd.org/content/dam/oecd/en/publications/reports/2023/12/pensions-at-a-glance-2023_4757bf20/678055dd-en.pdf.

[5] (2002). Active ageing: a policy framework. https://extranet.who.int/agefriendlyworld/wp-content/uploads/2014/06/WHO-Active-Ageing-Framework.pdf.

[6] Berkman LF, Kawachi I, Glymour MM (2014). Social Epidemiology.

[7] Edlin G, Golanty E (2012). Health & Wellness.

[8] Berkman LF, Glass T, Brissette I, Seeman TE (2000). From social integration to health: Durkheim in the new millennium. Soc Sci Med.

[9] Blazer DG (1982). Social support and mortality in an elderly community population. Am J Epidemiol.

[10] Levasseur M, Desrosiers J, Noreau L (2004). Is social participation associated with quality of life of older adults with physical disabilities?. Disabil Rehabil.

[11] Sayin Kasar K, Karaman E (2021). Life in lockdown: social isolation, loneliness and quality of life in the elderly during the COVID-19 pandemic: a scoping review. Geriatr Nurs.

[12] Tomioka K, Kurumatani N, Hosoi H (2017). Age and gender differences in the association between social participation and instrumental activities of daily living among community-dwelling elderly. BMC Geriatr.

[13] Liu AQ, Besser T (2003). Social capital and participation in community improvement activities by elderly residents in small towns and rural communities. Rural Sociol.

[14] Rabelo DF, Neri AL (2015). The household arrangements, physical and psychological health of the elderly and their satisfaction with family relationships. Rev Bras Geriatr Gerontol.

[15] Bernard M, Phillips J (2000). The challenge of ageing in tomorrow’s Britain. Ageing Soc.

[16] Lelkes O (2013). Happier and less isolated: internet use in old age. J Poverty Soc Justice.

[17] Bobillier Chaumon ME, Michel C, Tarpin Bernard F, Croisile B (2014). Can ICT improve the quality of life of elderly adults living in residential home care units? From actual impacts to hidden artefacts. Behav Inform Technol.

[18] Wu YH, Damnée S, Kerhervé H, Ware C, Rigaud AS (2015). Bridging the digital divide in older adults: a study from an initiative to inform older adults about new technologies. Clin Interv Aging.

[19] Castilla D, Botella C, Miralles I (2018). Teaching digital literacy skills to the elderly using a social network with linear navigation: a case study in a rural area. Int J Hum Comput Stud.

[20] Oh SS, Kim KA, Kim M, Oh J, Chu SH, Choi J (2021). Measurement of digital literacy among older adults: systematic review. J Med Internet Res.

[21] Kawaguchi K, Nakagomi A, Ide K, Kondo K (2024). Effects of a mobile app to promote social participation on older adults: randomized controlled trial. J Med Internet Res.

[22] Althoff T, Jindal P, Leskovec J Online actions with offline impact: how online social networks influence online and offline user behavior.

[23] Cao L, Chongsuvivatwong V, McNeil EB (2022). The sociodemographic digital divide in mobile health app use among clients at outpatient departments in Inner Mongolia, China: cross-sectional survey study. JMIR Hum Factors.

[24] Lin T, Guo W, Li Y, Guo X, Bai X, Min R (2024). Geographical accessibility of medical resources, health status, and demand of integrated care for older people: a cross-sectional survey from Western China. BMC Geriatr.

[25] Shaw LH, Gant LM (2002). In defense of the internet: the relationship between internet communication and depression, loneliness, self-esteem, and perceived social support. Cyberpsychol Behav.

[26] Morahan-Martin J, Schumacher P (2003). Loneliness and social uses of the internet. Comput Human Behav.

[27] Parker JDA, Taylor RN, Eastabrook JM, Schell SL, Wood LM (2008). Problem gambling in adolescence: relationships with internet misuse, gaming abuse and emotional intelligence. Pers Individ Dif.

[28] Ballarotto G, Volpi B, Marzilli E, Tambelli R (2018). Adolescent internet abuse: a study on the role of attachment to parents and peers in a large community sample. Biomed Res Int.

[29] Gencer SL, Koc M (2012). Internet abuse among teenagers and its relations to internet usage patterns and demographics. J Educ Technol Soc.

[30] Greydanus DE, Greydanus MM (2012). Internet use, misuse, and addiction in adolescents: current issues and challenges. Int J Adolesc Med Health.

[31] Unlimited (LTE) plan [Web page in Korean]. Korea Telecom (KT) Corporation.

[32] (2023). Korea Media Panel user guide, edition 2023 [Web page in Korean]. Korea Information Society Development Institute.

[33] Finlay JM, Kobayashi LC (2018). Social isolation and loneliness in later life: a parallel convergent mixed-methods case study of older adults and their residential contexts in the Minneapolis metropolitan area, USA. Soc Sci Med.

[34] (2021). 2020 Korea Media Panel Survey [Report in Korean]. https://www.kisdi.re.kr/report/view.do?arrMasterId=3934581&artId=558016&key=m2101113024973&masterId=3934581.

[35] Dehi Aroogh M, Mohammadi Shahboulaghi F (2020). Social participation of older adults: a concept analysis. Int J Community Based Nurs Midwifery.

[36] Townsend BG, Chen JTH, Wuthrich VM (2021). Barriers and facilitators to social participation in older adults: a systematic literature review. Clin Gerontol.

[37] Levasseur M, Cohen AA, Dubois MF (2015). Environmental factors associated with social participation of older adults living in metropolitan, urban, and rural areas: the NuAge study. Am J Public Health.

[38] Katagiri K, Kim JH (2018). Factors determining the social participation of older adults: a comparison between Japan and Korea using EASS 2012. PLoS ONE.

[39] Media statistics portal [Web page in Korean]. Korea Information Society Development Institute.

